# Discovery and characterization of an immunogenic neoantigen in a patient with metastatic triple negative breast cancer

**DOI:** 10.1186/2051-1426-3-S2-P284

**Published:** 2015-11-04

**Authors:** Yasmine Assadipour, Stephanie Goff, Jessica S Crystal, Todd D Prickett, Jared J Gartner, Robert Somerville, Hui Xu, Mary Black, Isaac Kriley, Paul F Robbins, Steven A Rosenberg, Steven Feldman

**Affiliations:** 1 George Washington University, Washington, DC, USA; 2 Surgery Branch, NIH/NCI, Bethesda, MD, USA; 3 NCI/NIH, Rutgers Robert Wood Johnson Medical School, New Brunswick, NJ, USA; 4 NCI/NIH, Bethesda, MD, USA; 5 Surgery Branch/National Cancer Institute/National Institutes of Health, Bethesda, MD, USA; 6 NIH/NCI, Bethesda, MD, USA

## 

A growing body of evidence suggests that successful clinical immunotherapy may depend on mutation-specific T cell responses. Using whole-exome and RNA sequencing of a resected metastatic deposit from a patient with triple-negative (ER-, PR-, Her2 non-amplified) breast cancer, we identified 72 non-synonymous mutations. Using previously published methods of autologous antigen presentation[1], we identified a novel non-synonymous mutation in a regulator of the Notch signaling pathway, *RBPJ* (recombination signal binding protein for immunoglobulin kappa J region), that encodes a neoantigen specifically recognized by autologous CD4+ tumor infiltrating lymphocytes. Antigen recognition was restricted by the Class II DR locus. Deep sequencing or PCR of all 16 metastases collected at autopsy revealed that the mutation was ubiquitously present in all samples. To the best of our knowledge, this represents the first report of an immunogenic mutation in breast cancer.
